# Targeting Injectable Hydrogels: The Role of Diphenylalanine Peptide Derivative in the Gelation Dynamics of Pluronic^®^ F127 †

**DOI:** 10.3390/polym17070930

**Published:** 2025-03-29

**Authors:** Vasile Robert Gradinaru, Maria Bercea, Luiza Madalina Gradinaru, Alexandru Puiu, Alexandra Lupu, Brindusa Alina Petre

**Affiliations:** 1 Faculty of Chemistry, Alexandru Ioan Cuza University of Iasi, 11 Carol I Bd., 700506 Iasi, Romania; puiualex162@gmail.com (A.P.); brindusa.petre@uaic.ro (B.A.P.); 2 “Petru Poni” Institute of Macromolecular Chemistry, 41-A Grigore Ghica Voda Alley, 700487 Iasi, Romania; bercea@icmpp.ro (M.B.); gradinaru.luiza@icmpp.ro (L.M.G.); lupu.alexandra@icmpp.ro (A.L.); 3 Center of Fundamental Research and Experimental Development in Translational Medicine (TRANSCEND), Regional Institute of Oncology, General Henri Mathias, No. 2–4, 700483 Iasi, Romania

**Keywords:** injectable hydrogel, Pluronic^®^ F127, peptide, hydrophobic interactions, shear thinning, self-healing

## Abstract

The fluorenyl methyl oxycarbonyl phenylalanyl-phenylalanine methyl ester (Fmoc-Phe-Phe-Ome) was synthetized using the liquid phase synthesis strategy. This derivative was separated by hydrophobic interaction chromatography, its purity was analyzed by RP-HPLC and it was characterized by mass spectrometry. This extremely hydrophobic peptide conjugate was incorporated into aqueous solutions of Pluronic^®^ F127 at low temperatures (below 10 °C). The temperature induced sol–gel transition was investigated by rheological measurements. A delay of the sol–gel transition, caused by the presence of low concentrations of Fmoc-Phe-Phe-Ome (up to 1%), enables better control of the gelation process. The viscoelastic properties of hybrid networks were investigated at 37 °C in different shear conditions. The Pluronic/peptide systems reported herein provide promising alternatives for developing innovative injectable gels as suitable platforms in cancer treatment.

## 1. Introduction

Peptides play a fundamental role in a plethora of living activities and are primarily involved, either directly or indirectly, in many physiological processes. Peptides have a wide range of customizable structures since they are made of amino acids in various configurations joined by amide bonds. Thus, based on the self-assembly behavior of peptides, peptide-based nanomaterials with a variety of morphologies can be designed and produced. Additionally, the ability to customize the side chains of amino acids offers multiple possibilities to design self-assembled materials for a variety of biomedical and engineering applications since the self-assembly behavior of peptides is typically triggered by internal or external environmental stimuli [1,2,3,4].

9-fluorenylmethoxycarbonyl-phenylalanyl-phenylalanine or Fmoc-Phe-Phe has been intensively studied over the past two decades. Β-Amyloid peptide, a well-known peptide that plays a crucial role in the pathology of Alzheimer’s disease, has a similar recognition characteristic motif (Phe-Phe) that is considered to be prone to aggregation. The presence of 9-fluorenylmethoxycarbonyl (Fmoc moiety) attached to dipeptide confers self-assembling and amphiphilic properties. This simple protected dipeptide can readily self-assemble to generate a biocompatible hydrogel [5]. The network formation is triggered by a combination of π–π stacking (enhanced by Fmoc moiety), and hydrophobic and hydrogen bonding interactions. Fmoc-Phe-Phe gelation can be initiated deploying one of the following methodologies: pH exchange, solvent or temperature switch, and catalytic assisted processes [6]. In a recent study, co-assembly of Fmoc-Phe-Phe dipeptides with trivalent metal ions, in aqueous condition, was investigated using ATR-IR, EPR spectroscopy and rheology [7]. One advantage of using Fmoc-Phe-Phe as a component of various supramolecular platforms consists in its rapid kinetics of hydrogels formation and mechanical strength [8].

Co-assembly of Fmoc-Phe-Phe with other components, including natural and synthetic polymers, might impact mechanical properties, including hydrogel stability, biocompatibility and other important characteristics. A hybrid hydrogel was obtained by self-assembly of Fmoc-Phe-Phe with alginate in solution followed by crosslinking of alginate assisted by calcium ions [9]. Strong hybrid hydrogel formation was induced by molecular crowding and hydrophobic effects when poly(ethylene glycol) and Fmoc-Phe-Phe were joined in an aqueous environment [10].

With regard to the ongoing aromatic interactions between the main constituents, doxorubicin entrapment into Fmoc-Phe-Phe hydrogels facilitated controlled drug release [11]. A hydrogel based on S-nitroso-N-acetyl-penicillamine and Fmoc-Phe-Phe was used to ameliorate ischemia [12]. Fmoc-dipeptides were also able to support chondrocytes cell culture in 2D and 3D formats [13]. Fmoc-Phe-Phe physical incorporation in alginate was performed in order to obtain a scaffold for bone regeneration [14]. The growth of encapsulated macrophage cells was supported by hydrogels with enhanced printability that contained Fmoc-Phe-Phe and crosslinked alginate [15]. Earlier studies emphasized that the materials containing phenylalanine dipeptide can carry the oligonucleotides into cells [16] and inhibit tumor cell growth [17,18]. Doxorubicin-loaded Fmoc-Phe-Phe nanogels are able to internalize into leukemic cells, influencing their viability [19].

Pluronic^®^ F127 (PL) is a versatile amphiphilic copolymer that self-assembles in aqueous environments like micelles or a network of polymicelles organized as large-scale structures with complex rheological behavior [20,21,22]. PL gels represent excellent matrices used for incorporating various active principles [23,24], presenting high transfection efficiency and reduced cytotoxicity [17].

Thus, Pluronic-based gels are currently used in various applications as appropriate matrices for ocular, nasal, transdermal or subcutaneous drug delivery [24,25]. However, the application of a single PL-based gel is limited by its relatively fast dissolution under physiological conditions [25] and poor antimicrobial properties [26]. Several potential alternatives to this issue have been explored, either by modifying the copolymer structure [27] or incorporating other compounds, such as polysaccharides [28,29,30] and bioactive compounds [23].

It was shown that the phenylalanine dipeptides interact with a PL derivative (having the hydroxyl terminal groups oxidized to aldehyde group) via Schiff bonds and the obtained self-assembling materials have proved to have promising applications as tumor-targeting carriers [18]. Under acidic conditions, the Schiff bonds are broken and favor a targeted drug delivery, for example, to breast cancer cells [18]. The peptide with two phenylalanine moieties favors the material aggregation through the steric hindrance [31].

In the present paper, a new phenylalanine-based derivative was synthetized, characterized and incorporated into Pluronic^®^ F127 gels. The rheological investigations have shown that a low amount of peptide (up to 1%) influences the sol–gel transition and gel properties at 37 °C.

## 2. Experimental Section

### 2.1. Materials

L-phenylalanine and Fmoc-phenylalanine were purchased from Carl Roth Gmbh (Karlsruhe, Germany) and Fluka (Sigma-Aldrich Chemie, Steinheim, Germany), respectively. Trimethyl chlorosilane, 1-hydroxybenzotriazole, hydrochloric acid (25%) and dichloromethane were procured from Merck (Darmstadt, Germany). Dicyclohexylcarbodiimide (DCC) was acquired from Acros Organics (Geel, Belgium). Trifluoroacetic acid was purchased from Merck (Hohenbrunn, Germany). Methanol and acetonitrile (I), both HPLC grade, were obtained from Carl Roth (Karlsruhe, Germany) and Honeywell Riedel-de Haen™ (Seelze, Germany). Ethyl acetate and sodium carbonate were purchased from Reactivul (Bucharest, Romania). Triethylamine, sodium carbonate and sodium sulfate (anhydrous), dihydroxybenzoic acid as well as super-DHB, a mixture of 2,5-dihydroxybenzoic acid (2,5-DHB) and 2-hydroxy-5-methoxybenzoic acid (9:1, *w*/*w*), were procured from Merck (Darmstadt, Germany). Sodium chloride was obtained from Lach-Ner (Neratovice, Czech Republic).

Pluronic^®^ F127 (PL) was purchased from Sigma-Aldrich Co. (Taufkirchen, Germany). This triblock copolymer with molecular weight of 12.6 kg/mol contains poly(ethylene oxide) (PEO) and poly(propylene oxide) (PPO) blocks in its structure, i.e., EO_100_-PO_65_-EO_100_.

### 2.2. Methods

#### 2.2.1. Syntheses of Phenylalanine Methyl Ester and Fmoc-Phenylalanyl-Phenylalanine Methyl Ester

Phenylalanine methyl ester (Phe-Ome, 3 mmol) was obtained from its corresponding amino acid using trimethyl chlorosilane (7.5 mmol) as methylating agent [32]. The reaction was performed at room temperature (for 20 h) using methanol as a solvent.

**Scheme 1:**
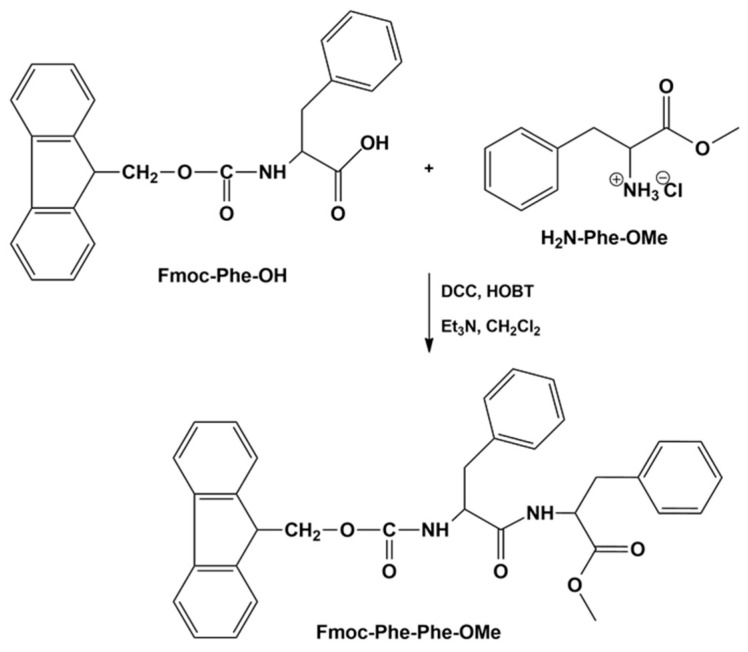
The coupling reaction of fluorenyl methyl oxycarbonyl phenylalanine with phenylalanine (methyl ester), using DCC/HOBt as coupling agents.

The liquid phase synthesis of Fmoc-Phe-Phe-Ome was achieved using Fmoc-Phenylalanine (Fmoc-Phe-OH) and Phe-Ome. This reaction was performed for 48 h at room temperature in dichloromethane using dicyclohexylcarbodiimide (DCC)/1-Hydroxybenzotriazole (HOBt) system in triethyl amine (Scheme 1). After the reaction, the mixture was evaporated, and the resulted solid was suspended in ethyl acetate and filtered. The resulted filtrate was washed/separated three times with 1 M HCl, two times with 5% NaCl, three times with 1 M sodium carbonate and again two times with 5% NaCl solution. The organic layer was dried using sodium sulphate anhydrous and later filtered and evaporated.

#### 2.2.2. UV–Vis Spectroscopy

All absorption spectra of free phenylalanine, Fmoc-protected (Fmoc-Phe-OH) and purified Fmoc-dipeptide were acquired using a Libra UV–Vis spectrophotometer (Biochrome, Cambridge, UK). Each diluted solution was placed in a 1 cm quartz cell, and the corresponding spectrum was registered using a spectral range of 200–400 nm (scanning speed of 2649 nm/min in 1 nm steps).

#### 2.2.3. Reverse-Phase High-Performance Liquid Chromatography (RP-HPLC)

Both esterification and coupling reactions were monitored using a UHPLC system (DionexUltiMate 3000 from Thermo Scientific, Waltham, MA, USA). The second reaction, targeting Fmoc-dipeptide methyl ester, was followed by HPLC system using a Vydac RP-C18 column (250 mm × 4.6 mm, 5 µm silica, pores size 300 Å) from Waters (Milford, MA, USA) and a DAD detection system. Three different wavelengths were selected for detection: 220 nm (characteristic to the amide and urethane bond), 260 nm and 320 nm (specific to the aromatic and fluorenyl moieties). The column chamber was thermostated at 25 °C for all runs. A mixture of two solvents was used as a mobile phase: 0.1% trifluoroacetic acid (TFA) aqueous solution, denoted A, and 80% acetonitrile (I) in 0.1% TFA, denoted B. Before its usage, the solvent mixture underwent a degassing process for 45 min in an ultrasonic bath. The flow rate of 1.0 mL/min was constantly maintained during each separation. All samples were applied in 5% B (a volume of 20–80 µL was automatically injected by autosampler) and, after 2 min, a linear gradient elution was applied (5–100% B for 23 min) during separation. Then, mobile phase concentration was maintained at a higher value (100% B) followed by a drop to an initial one (5% B) and an equilibration final step (for each step, a 4 min timeframe was established).

#### 2.2.4. ^1^H and ^13^C-NMR Spectroscopy

The NMR spectra were registered using a Bruker Avance NEO spectrometer (400 MHz, Bruker Switzerland AG, Fällanden, Switzerland) with a 5 mm direct detection probe. DMSO-d6 was used as deuterated solvent. All chemical shifts were expressed as δ values (ppm) and referenced as a function of employed solvent peaks (^1^H: 2.50 ppm; ^13^C: 39.5 ppm).

#### 2.2.5. MALDI-ToF/ToF Mass Spectrometry

The mass spectra were recorded using a matrix-assisted laser desorption/ionization time-of-flight mass spectrometer Bruker Ultraflex (MALDI ToF/ToF) from Bruker Daltonics (Bremen, Germany). The measurements were handled in positive reflectron mode and a super-DHB (9:1; *w*/*w*) mixture consisting of 2,5-dihydroxy-benzoic acid: 2-hydroxy-5-methoxy-benzoic acid was used as a matrix. The crude and pure peptide derivate was diluted into a mixture I: 0.1% TFA (aqueous) at a 3:2 ratio. Each investigated sample was co-crystallized by directly mixing it at a 1:1 ratio with a super-DHB solution on a 348-spot MALDI target plate using the sandwich method.

#### 2.2.6. Rheological Measurements

The rheological tests were performed by using a MCR 302 rheometer (Anton Paar, Graz, Austria) equipped with plane–plane geometry (diameter of 25 mm and gap of 0.5 mm) and a Peltier device for rigorous temperature control.

For each hydrogel sample, the viscoelastic parameters were monitored during amplitude sweep tests at oscillation frequency (ω) of 10 rad/s, or frequency sweep tests performed at strain amplitude (γ) of 1% (value that is in the linear viscoelastic domain).

The sol–gel transition was evidenced using solutions stored in refrigerator and introduced prior to each test into the geometry of rheometer, thermostated at 5 °C. The following viscoelastic parameters were determined in dynamic conditions: elastic (*G*′) and viscous (*G*″) moduli and loss tangent (tan*δ*). The gelation temperature was determined by applying a controlled heating rate (1 °C/min) at constant frequency of oscillation (*ω* = 10 rad/s) and strain amplitude (γ = 1%). The gelation time at 37 °C was determined by monitoring the viscoelastic parameters as a function of time (*ω* = 10 rad/s, γ = 1%) when the temperature was suddenly changed from 5 °C to 37 °C.

The viscosity (η) was determined as a function of the shear rate ($\dot{\gamma }$) in stationary continuous shear conditions, for 0.01 s^−1^ < $\dot{\gamma }$ < 1000 s^−1^. The yield stress (τ_o_) was determined in amplitude sweep tests as a measure of the shear force that can be applied to hydrogel before it starts to flow.

The self-healing ability was monitored in oscillatory shear for *ω* = 10 rad/s, using three strain step tests, when the strain values are changed every 300 s from γ = 1% (which is in the linear domain of viscoelasticity) to high strain values (which are in the nonlinear domain of viscoelasticity, i.e., γ of 50%, 100%, 300%, 500% and 1000%) and then again to the previous low strain value (1%).

## 3. Results and Discussion

### 3.1. Characterization of Phenylalanine Methyl Ester (Phe-Ome)

The presence of resulting ester was confirmed by thin layer chromatography, ^1^H-NMR and ^13^C-NMR. The chemical shifts assigned for aromatic protons of phenylalanine before (7.20–7.30 ppm) and after esterification (7.23–7.35 ppm) were noticed in ^1^H-NMR spectra. Methylene moiety protons have chemical shifts at 2.79–3.16 and 3.07–3.21 ppm. Methyl group protons can be easily assessed at 3.63 ppm and are in frame with the previously reported data [33]. Supplementarily, ^13^C-NMR spectrum displays a chemical shift at 127.31–129.43 ppm for aromatic carbons, a characteristic signal at 37.05 ppm to methylene, and two signals at 52.59 and 53.23 ppm corresponding to newly formed O-CH_3_ and alpha carbon groups.

### 3.2. Separation of Fmoc-Phenylalanyl-Phenylalanine Methyl Ester (Fmoc-Phe-Phe-Ome)

Since the targeted crude product was less soluble in 5% B (starting liquid phase for HPLC separation), a C18-reverse phase cartridge was used for protected dipeptide separation starting with 50% MeOH (aqueous solution) and by increasing the organic solvent content. All fractions were analyzed by UV–Vis spectroscopy in the spectral domain of 200–340 nm. More precisely, the product was separated at the highest MeOH concentration and displayed a characteristic maximum at 269 nm (attributed to phenyl moiety) and two adjacent shoulders situated at 259 and 282 nm. Besides, two signals with moderate intensity at 292 and 302 nm attributed to fluorenyl protecting group are also a fingerprint of the desired compound. The purified product is distinguished by an absorption ratio A_269_/A_302_ of 2.35.

**Figure 1:**
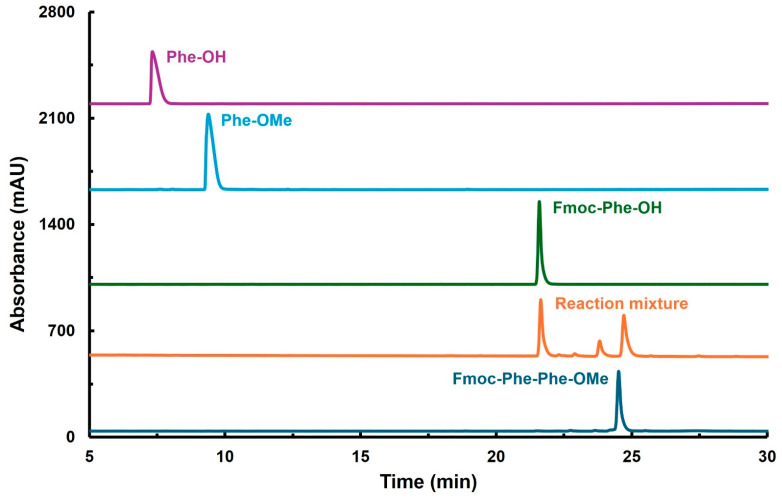
HPLC separation profiles of phenyl alanine (20 µg Phe-OH; dark violet), phenyl alanine methyl ester (40 µg Phe-Ome; dark cyan), N-protected amino acid (5 µg Fmoc-Phe-OH; dark green), reaction mixture (20 µL 5.2% mixture in 5% B; dark orange) and Fmoc-dipeptide methyl ester (Fmoc-Phe-Phe-Ome fraction from C18-Sepack column eluted at 100% MeOH; steel blue). The absorbance was monitored at 220 nm.

All reactants, the reaction mixture and above collected fractions (including those containing purified protected dipeptide) were analyzed by HPLC at three different wavelengths (220, 260 and 320 nm). The retention times for phenyl alanine (Phe-OH), phenyl alanine methyl ester (Phe-Ome), N-protected amino acid (Fmoc-Phe-OH) and Fmoc-dipeptide methyl ester were 7.32, 9.40, 21.60 and 24.52 min (Figure 1).

A slight retention time shift (the”prod’c) was eluted 0.19 min earlier) was noticed due to the double MeOH final concentration (10% MeOH) of the investigated purified sample as compared with reaction mixture. Thus, the pure compound was eluted lately at a concentration of 78.4% I. Both starting agents, Phe-Ome and Fmoc-Phe-OH, were eluted at I concentrations of 28.5 and 68.8%, respectively.

### 3.3. Characterization of Fmoc-Phe-Phe-Ome by Mass Spectrometry

**Figure 2:**
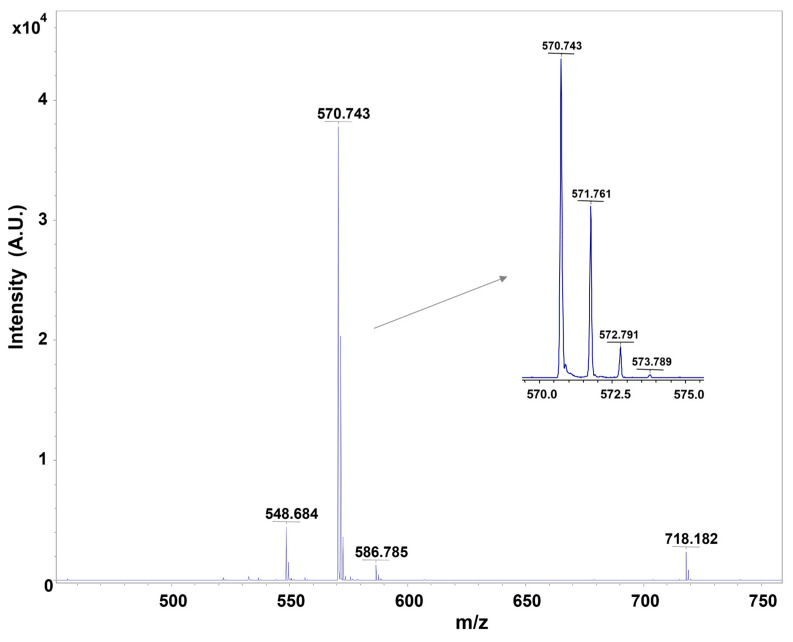
MALDI-ToF MS of monoprotonated eprotected dipeptide and its corresponding natrium and potassium adducts. Insert: monoisotopic distribution of parent ions.

The raw and purified reaction products were analyzed by MALDI-ToF/ToF using super DHB as a matrix. The molecular ion displays a signal at *m*/*z* 548.684. The peptides corresponding to natrium and potassium adducts were observed at *m*/*z* 570.743 and 586.785, respectively, with the monoisotopic distribution of natrium adduct displayed in Figure 2. More precisely, four isotopomers were observed at 570.743, 571.761, 572.791 and 573.789. Notably, a low-intensity signal at *m*/*z* 718.182 was also detected in both the raw reaction mixture and the purified fraction, corresponding to the presence of the protected tripeptide (Fmoc-Phe-Phe-Phe-Ome) as a side product.

The MS/MS of the dipeptide derivate was further analyzed by LIFT-ToF/ToF [34]. The compound contains one peptide bond, one urethane moiety and an ester group. Thus, a relatively simple fragmentation pattern was expected. A fragment with moderate intensity was observed at *m*/*z* of 517.56 (Figure S1) and was attributed to a positively charged R-C≡O^+^ ion, resulting from the loss of an -Ome moiety. A high-intensity signal at *m*/*z* 327.323, characteristic of the protonated ^+^NH_3_-Phe-Phe-Ome form, was observed and assigned to a fragment generated by the cleavage of the amide bonds in urethane moiety (Figure S2). The Y’ ion, retaining the positive charge on the C-terminal fragment, displayed the highest intensity in the MS/MS spectra, as illustrated in Figure S2. Further fragmentation of the parent ion led to the loss of a CO_2_ molecule forming a fluorenyl carbocation [35], with a distinct signal at *m*/*z* 179.179 (Figure S1). Additionally, an adjacent low-intensity signal at *m*/*z* of 164.136 was attributed to 9-fluorenylidene diradical, generated by the loss of methyl cation. Conversely, the peptide bond proved to be relatively stable, as indicated by the low-intensity signal of the phenyl-alanine methyl ester at *m*/*z* 180.204. Moreover, a b-type ion was detected at *m*/*z* 371.376, and the difference between this signal and that at *m*/*z* 180.204 confirms the presence of phenylalanine moiety situated in the middle of the structure. Therefore, the MS/MS approach successfully validated the chemical structure of the product and the fragmentation species, as depected in Scheme 1 and Figure S1.

### 3.4. Rheological Investigation

For the rheological investigations, samples of 10, 15 and 20% PL were prepared, and different contents of Fmoc-Phe-Phe-Ome were added: 0.1, 0.5 and 1%. Our major goal was to obtain stable hybrid gel formulations at 37 °C, with shear thinning behavior and self-healing properties.

#### 3.4.1. The Influence of Fmoc-Phe-Phe-Ome on the PL Gelation Induced by the Temperature Increase

**Figure 3:**
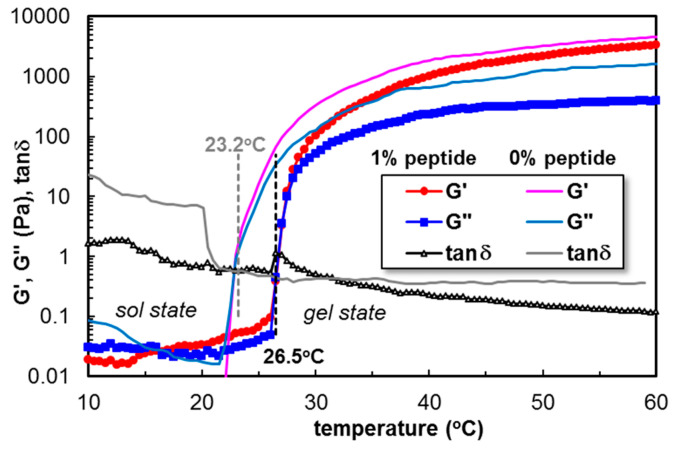
The variation of viscoelastic parameters by increasing the temperature with a heating rate of 1 °C/min for systems containing 15% PL in the presence/absence of 1% Fmoc-Phe-Phe-Ome (ω = 1 rad/s, γ = 1%).

The samples were stored at 4–5 °C (in refrigerator) prior to testing and placed into the measuring geometry thermostated at 5 °C. The viscoelastic parameters were monitored at a range of temperatures situated between 5 °C and 80 °C. Figure 3 illustrates, as an example, the behavior of 15% PL in aqueous solution compared to the same sample in which 1% Fmoc-Phe-Phe-Ome was incorporated. Only the data between 10 °C and 60 °C were considered relevant for the present study.

**Figure 4:**
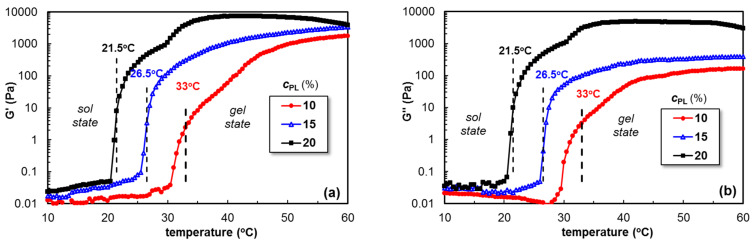
The dependences of (**a**) G′ and (**b**) G″ on temperature for PL samples of different concentrations in the presence of 1% Fmoc-Phe-Phe-OMe (heating rate of 1 °C/min, ω = 1 rad/s, γ = 1%).

**Figure 5:**
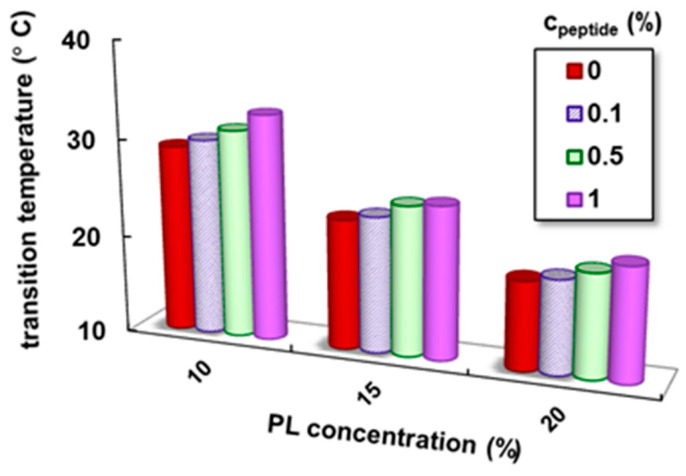
The influence of peptide content on the sol–gel transition temperature of PL samples.

At low temperatures, the PL aqueous systems are in sol state (with predominantly viscous behavior), allowing a homogeneous loading of Fmoc-Phe-Phe-Ome. By increasing the temperature, the sol–gel transition occurs and, in a narrow temperature range, a network with solid-like behavior is formed. The gelation point (T_sol–gel_) is considered as the temperature for which G′ = G″. In the presence of 1% peptide, the fluid is more structured In both the sol and gel states (a decrease of tanδ value) and the transition point is slightly shifted to higher temperatures (from 23.2 °C to 26.5 °C). The increase in the PL concentration considerably diminishes the T_sol–gel_ value (Figure 4 and Figure 5). The triblock copolymer is able to self-assemble above the critical micellization concentration (cmc), which strongly decreases as the temperature is raised. At low temperatures, the hydration layers of PEO surrounding PL macromolecules favor dissolution of the copolymer. As the temperatures increase, the hydrogen bonds established between water hydrophilic PEO segments become weaker, whereas the hydrophobic interactions among the PPO domains become predominant. In these conditions, micelles and polymicelles are spontaneously formed, generating a network structure above T_sol–gel_ when a sharp increase in the viscoelastic parameters is registered.

The sol–gel transition temperature is more sensitive to peptide content at lower PL concentrations (10%) as compared with more concentrated (15% and 20%) PL-based systems (Figure 5). This can be explained by the formation of PL–peptide complexes at lower concentrations and immobilized Fmoc-Phe-Phe-OMe into the polymicellar structures at higher concentrations. Thus, by adjusting the PL concentration, the hydrodynamic size of hybrid nanostructures and aggregates can be varied in order to achieve a suitable drug delivery system [36].

#### 3.4.2. The Network Formation at Physiological Temperature

**Figure 6:**
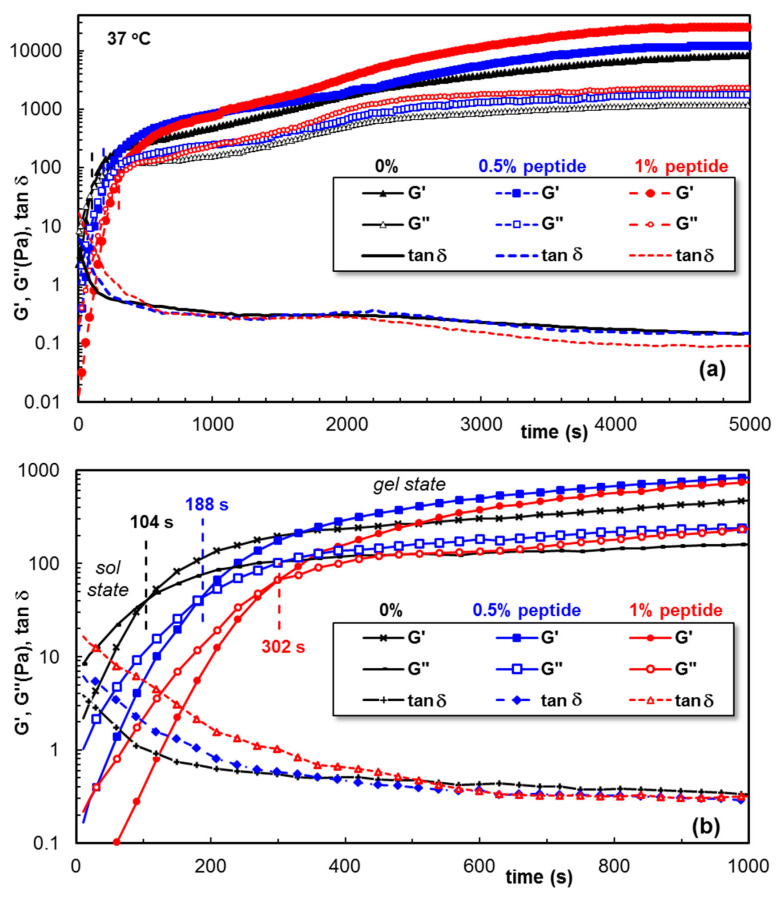
The gelation kinetics at 37 °C through the evolution of the viscoelastic parameters for 15% PL in the presence of different Fmoc-Phe-Phe-OMe concentrations (**a**) for 5000 s and (**b**) detailed image showing the evidence of the sol-gel transition during the first 1000 s of heating at 37 °C (ω = 1 rad/s, γ = 1%).

The PL-based samples (which were previously stored in refrigerator) were introduced into the geometry of the rheometer at 5 °C at the beginning of the experiment; then, the temperature was suddenly switched to 37 °C. The viscoelastic parameters were determined at a constant oscillation frequency of 1 rad/s and strain amplitude of 1% (Figure 6), and the network formation was monitored as a function of time in temperature conditions similar to physiological ones.

The sol–gel transition time is an important parameter for injectable hydrogels in tissue engineering applications. Generally, 300–600 s is considered as an optimal gelation time, required to manipulate the sample and administer it to the target site [36]. In the present study, the gelation time was considered as the time for which G′ = G″.

**Figure 7:**
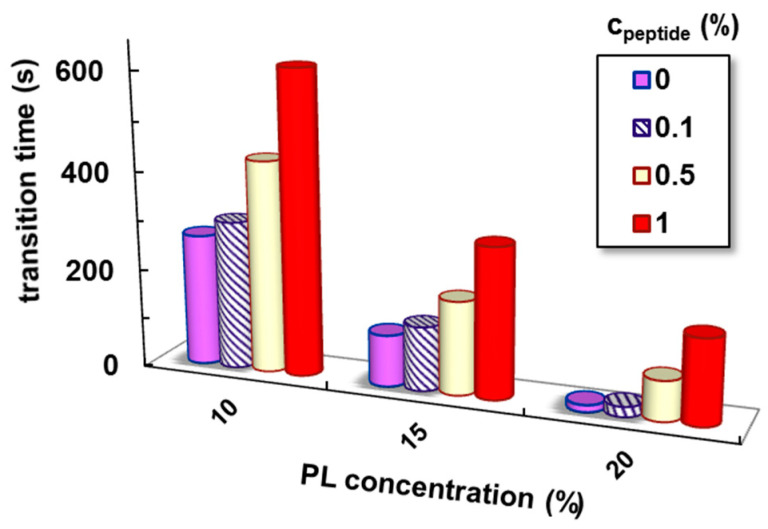
The gelation time at 37 °C for PL samples in the presence of different Fmoc-Phe-Phe-OMe concentrations (ω = 1 rad/s, γ = 1%).

For samples containing 20% PL, the sol–gel transition takes place very fast (within 20–30 s). The gelation time increases in the presence of peptide, reaching about 3 min for 1% Fmoc-Phe-Phe-OMe (Table 1). Lower PL concentrations (i.e., 15% and 10% PL) induced higher gelation times (Figure 7). A concentration of 15% for PL appears as a suitable concentration for PL in injectable gel formulations with an optimum sol–gel transition time (between 100 s and 300 s) and a gelation temperature that ranges from 21 °C to 33 °C (Figure 4).

Furthermore, the time required to reach the equilibrium state of the network structure increases from 5 to 10 min in the case of 20% PL-based samples to about 15 min for 15% PL and more than 1 h for samples containing 10% PL.

The micellar network structure progressively formed during the gelation of PL in the presence of Fmoc-Phe-Phe-OMe at 37 °C is influenced by the concentration of PL and peptide, as reflected by the values registered at equilibrium for various viscoelastic parameters (Table 1). Thus, the elastic and viscous moduli (G′ and G″) and the upper limit of strain for the linear viscoelastic range (γ_L_) increase as the PL concentration increases. The yield stress (τ_o_) is defined as the highest shear stress value for which the response to deformation is still elastic [37,38]. Therefore, for σ < σ_o_, the rest structure is still preserved, and for σ > σ_o_, the flow starts and parts of the macromolecular chains begin to orient along the flow direction. The characteristic gel parameters (G′, G″, γ_L_, τ_o_, η) increase with the peptide content (Table 1), suggesting that the intermolecular interactions are stronger in the presence of peptide. This behavior is similar to those observed for thermosensitive polyurethanes in the presence of an octapeptide having a sequence found in type IV collagen [39,40]. For indole-capped dipeptide, the G′ values depend on the preparation method: using the pH switch method, the gels present G′ around 3 × 10^5^ Pa, whereas for gels prepared by solvent switch or temperature methods, G′ values were below 10^4^ Pa [41]. Another thermoresponsive system of peptide amphiphile and poly(N-isopropylacrylamide-*co*-formyl phenyl acrylate), recently reported in [38], showed a mechanical stiffening response under the actions of temperature and external stress.

Gelation of Pluronic F127 aqueous solutions is attributed to a slow process of micelles arrangement into large-scale polymicellar structures [20,21], which takes place above the transition temperature. According to the rheological data obtained at 37 °C, the presence of Fmoc-Phe-Phe-OMe hydrophobic peptide increases the transition time (Table 1), and a stronger structure is generated in time. At raised PL concentrations (i.e., 20%), the micelle-like structures contain immobilized peptide molecules [42].

The main driving forces that influence the self-assembly of PL macromolecules are the intermolecular interactions. Thus, the hydrophobic/hydrophilic balance, which is dictated by the temperature change, hydrogen bonding, van der Waals forces or steric hindrance, determines the overall behavior [38,40,43,44]. The synergetic action of different interactions in Pluronic/peptide aqueous systems can be explored to generate networks with well-controlled properties for the development of new biomaterials for tissue engineering or therapeutic applications [29,38,45,46].

#### 3.4.3. The Shear Flow Behavior

**Figure 8:**
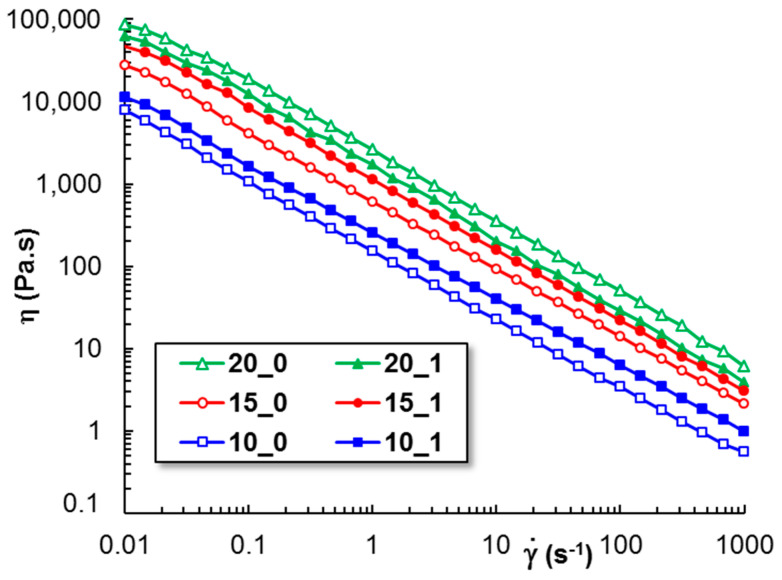
The shear viscosity as a function of shear rate for hydrogels of 10%, 15% and 20%, in the absence of peptide (open symbols, abbreviation ending with 0) and in the presence of 1% Fmoc-Phe-Phe-OMe (full symbols, abbreviation ending with 1) at temperature of 37 °C.

At physiological temperature, the PL-based gel formulations present shear thinning behavior. The viscosity (η) decreases as the shear rate ($\dot{\gamma }$) increases, varying as ${\dot{\gamma }}^{-0.80}$ ÷ ${\dot{\gamma }}^{-0.86}$ (Figure 8).

The thermosensitive gels present low viscosity in sol state during storage at low temperatures and they are easily administered in situ with a syringe or used for bioprinting, being able to create the required structure shape. In addition, the gels present pseudoplastic behavior; at high shear rates, the viscosity decreases by about three orders of magnitude. For PL concentrations of 10% and 15%, the shear viscosity increases in the presence of Fmoc-Phe-Phe-OMe. Also, the addition of the hydrophobic peptide increases the transition time (Figure 7, Table 1) and the network strength improves in time. For more concentrated PL gels (concentration of 20%), the shear viscosity decreases when the peptide is incorporated. The peptide immobilized into the hydrophobic core [42] changes the hydrophilic/hydrophobic balance, and the PL hydrogel tends towards phase separation when higher Fmoc-Phe-Phe-OMe content (above 1%) is incorporated.

#### 3.4.4. Self-Healing Behavior

**Figure 9:**
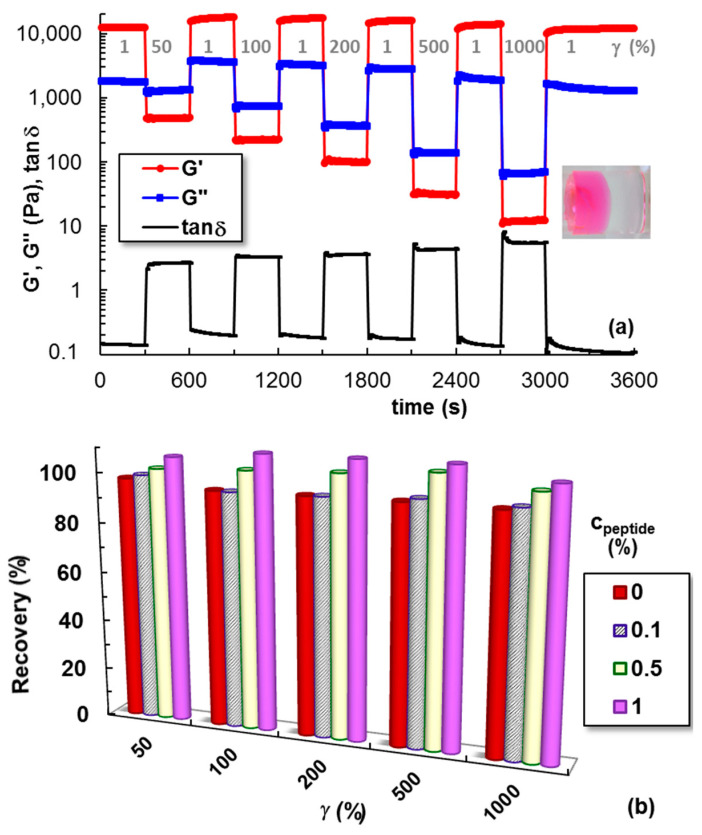
Self-healing behavior illustrated for samples containing 15% PL and different Fmoc-Phe-Phe-OMe concentrations (ω = 1 rad/s, 37 °C): (**a**) Five step strain cycles were applied to the sample that contain 15% PL and 1% peptide, by alternating small–high–small strain values. The small level of strain was always 1% and the high level of strain was increased successively: 1–50%; 2–100%; 3–200%; 4–500%; and 5–1000%. (**b**) The recovery degree for samples containing 15% PL and different Fmoc-Phe-Phe-OMe concentrations, after applying the five step strain cycles.

The self-healing ability of hydrogels—that is, the capacity to restore their original structure after they exhibited high deformations—is one of the most important characteristics required for injectable materials [23,27,29,36,37,43]. In the present study, the thixotropic behavior was monitored in oscillatory shear conditions. Figure 9a presents, as an example, the variation of viscoelastic parameters (G′, G″ and tanδ) as a function of time for the sample containing 15% PL and 1% peptide. The strain value was set firstly to a low strain value of 1% (in the linear domain of viscoelasticity); then, γ was successively changed to high values (50%, 100%, 200%, 400%, 500%, 700% and 1000%, which belong to nonlinear range of viscoelasticity), and the strain returns to its initial value of 1%.

The degree of recovery expresses the ability of the polymicellar structure to be rapidly reestablished after removing the external stress that determines a high deformation. The specific behavior of each sample can be depicted by evaluating the degree of structure recovery after each strain cycle:

(1) $$\mathrm{Recovery}\;(\%)=\frac{G\prime \;\mathrm{before}\;\mathrm{the}\;\mathrm{first}\;\mathrm{cycle}}{G\prime \;\mathrm{after}\;\mathrm{the}\;\mathrm{cycle}\;N}\times 100$$

The recovery degree of 15% PL samples after each cycle of deformation is given in Figure 9b. According to this analysis, the hydrogel samples that contain 1% peptide present structural integrity after subjecting them to several cycles of increasing γ. When the action of the external force is removed, the physical interactions are restored and, thus, a fast recovery of the network structure is registered.

The present investigation indicates that a suitable formulation obtains the sample of 15% PL incorporating 1% peptide that behaves as a soft and elastic gel (sol phase at low and ambient temperature and gel at physiological temperature, upon implantation at the target site). For this gel, we observed a high degree of structure recovery (Figure 9b).

The PL-based gels exhibit high yield stress values, which can reach hundreds of Pascals for 15% or 20% copolymer concentrations (Table 1), acting as viscoplastic fluids.

Gelation of Pluronic F127 aqueous solutions is attributed to a slow process of micelles arrangement into large-scale polymicellar structures [20,21], which takes place above the transition temperature. According to the rheological data, the presence of Fmoc-Phe-Phe-OMe hydrophobic peptide increases the transition time at 37 °C (Table 1), but, over time, a stronger structure is generated.

The amphiphilic feature of the Fmoc-derived dipeptide derivative reported here promotes the self-assemblage of Fmoc groups. Created microstructures by the Fmoc-dipeptides at the air/water boundary could only be directly seen in situ, courtesy of an optical technique known as Brewster Angle Microscopy [47]. Thus, the Fmoc-Phe-Phe dipeptide, having two aromatic amino acids as the well-studied β-amyloid peptide, could form a hydrogel. This supramolecular architecture is facilitated by an array of hydrophobic and hydrogen bonding interactions and π–π stacking interactions, respectively [48,49]. Aromatic moieties (attached to the N-terminal) could also enhance the self-assembly process of protected peptides, stabilize their conformations and generate new features due to the favorable contributions of the aromatic groups (such as π–π interactions) and hydrophobic interactions. Smith and coworkers have also emphasized that Fmoc-Phe-Phe has the capacity to self-assemble as cylindrical fibrils. These nanostructures are generated via π–π interconnected antiparallel β-sheets [50].

Another study reflected that Fmoc-Phe-Phe could be a promising starting compound that self-assembles into a versatile scaffold suitable for tissue engineering. The authors suggest a remarkable mechanical rigidity of the resultant hydrogel compared with other physically cross-linked ones. On the other hand, a direct correlation between peptide concentration and rheological behavior was noticed. Finally, it was previously revealed that this material may boost the attachment of Chinese Hamster Ovarian cells [51].

Two-dimensional and three-dimensional experiments reveal the capacity of Fmoc-Phe-Phe gel to support retention and growth of phenotype bovine chondrocytes. Dried Fmoc-Phe-Phe peptide hydrogels were distinguished by piezoelectricity when analyzed using piezoresponse force microscopy. Together with these electric characteristics, Fmoc-Phe-Phe-based gels were also recommended as useful instruments for applications requiring electrical stimuli (such as axonal regeneration) because of their biomimetic qualities. The balance between intra- and intermolecular contacts is directly connected to the Fmoc-Phe-Phe phase behavior [8].

**Scheme 2:**
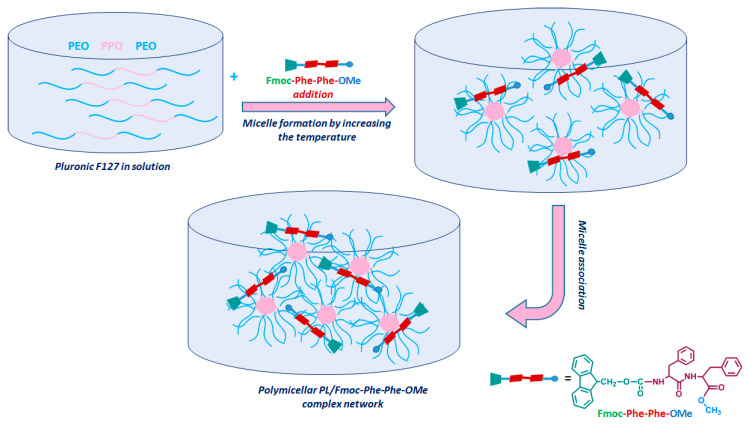
Representation of temperature-induced gelation of Pluronic F127 in the presence of Fmoc-Phe-Phe-OMe.

Scheme 2 is a concise illustration of the thermal induced micellization and gelation of PL in the presence of Fmoc-Phe-Phe-OMe. These complex networks may function as matrices for long-acting hydrophobic therapeutic agents [23,52,53].

## 4. Conclusions

The purpose of our study was to obtain hybrid hydrogels with shear thinning flow and self-healing properties. The fluorenyl methyl oxycarbonyl phenylalanyl-phenylalanine methyl ester (Fmoc-Phe-Phe-OMe) was synthetized using liquid phase synthesis strategy and further characterized. This protected dipeptide was included in aqueous solutions of Pluronic F127 at low temperatures (below 10 °C). As temperature increases, micelles are formed, generating a physically crosslinked network structure, which was investigated by rheology. It was observed that the temperature-induced gelation process and viscoelastic properties of hydrogels at 37 °C are sensibly influenced by the presence of dipeptide, especially at a moderate Pluronic concentration (around 15%). These systems present shear thinning properties; at high shear rates, the viscosity decreases by several orders of magnitude. By varying the concentrations of Pluronic F127 and of dipeptide, the system can be fine-tuned to generate thermosensitive gels with the tunable rheological response and stability required for injectable gels.

The hybrid polymer/peptide hydrogels are tailorable and versatile systems with complex rheological behavior that can be considered as suitable platforms in cancer treatment.

## Figures and Tables

**Table 1:** Some rheological characteristics determined for PL-based hydrogels.

c_PL_ (%)	c_peptide_ (%)	T_sol–gel_ (°C)	Sol–Gel Transition Time (s)	G′ * (Pa)	G″ * (Pa)	Tanδ *	γ_L_ * (%)	τ_o_ * (Pa)	η_100_ ** (Pa·s)
10	0	29.1	266	2795	269	0.0963	15.7	63.0	3.4
	0.1	30.0	300	2880	291	0.1011	15.2	64.3	3.5
	0.5	31.2	429	3081	402	0.1305	15.9	66.1	4.8
	1	33.0	615	3359	521	0.1551	16.5	68.5	6.3
15	0	23.2	104	8040	1200	0.1493	20.7	123	14.1
	0.1	23.8	129	8930	1371	0.1535	20.6	125	14.6
	0.5	25.3	188	10,100	1780	0.1762	25.4	137	16.9
	1	26.5	302	11,550	2281	0.1964	32.9	149	21.9
20	0	19.0	14	14,900	2940	0.1973	66.3	404	50.9
	0.1	19.5	20	14,946	3030	0.2027	67.4	399	43.2
	0.5	20.5	81	15,455	3021	0.1955	65.5	367	32.8
	1	21.5	173	16,050	2912	0.1814	63.2	352	28.3

* Determined in oscillatory shear conditions for hydrogels thermostated at 37 °C. ** Determined in continuous shear conditions at $\dot{\gamma }$ = 100 s^−1^ for hydrogels thermostated at 37 °C.

## Data Availability

The original contributions presented in this study are included in the article. Further inquiries can be directed to the corresponding author.

## Supplementary Materials

The following supporting information can be downloaded at: https://www.mdpi.com/article/10.3390/polym17070930/s1, Figure S1: MS/MS spectrum of fragmented protected peptide; Figure S2: MS/MS Fmoc-Phe-Phe-OMe fragmentation pattern; Figure S3: The viscoelastic moduli as a function of strain for 15% PL at 37 °C.
