# Supplementary material for: Targeting Injectable Hydrogels: The Role of Diphenylalanine Peptide Derivative in the Gelation Dynamics of Pluronic® F127
Source: Polymers (Basel). 2025 Mar 29;17(7):930. doi: 10.3390/polym17070930 (PMC11991411; doi:10.3390/polym17070930)
Supplement: Supplementary file 1 [file polymers-17-00930-s001.zip › polymers-3531280-supplementary.pdf]

polymers

MDPI

Supplementary Materials

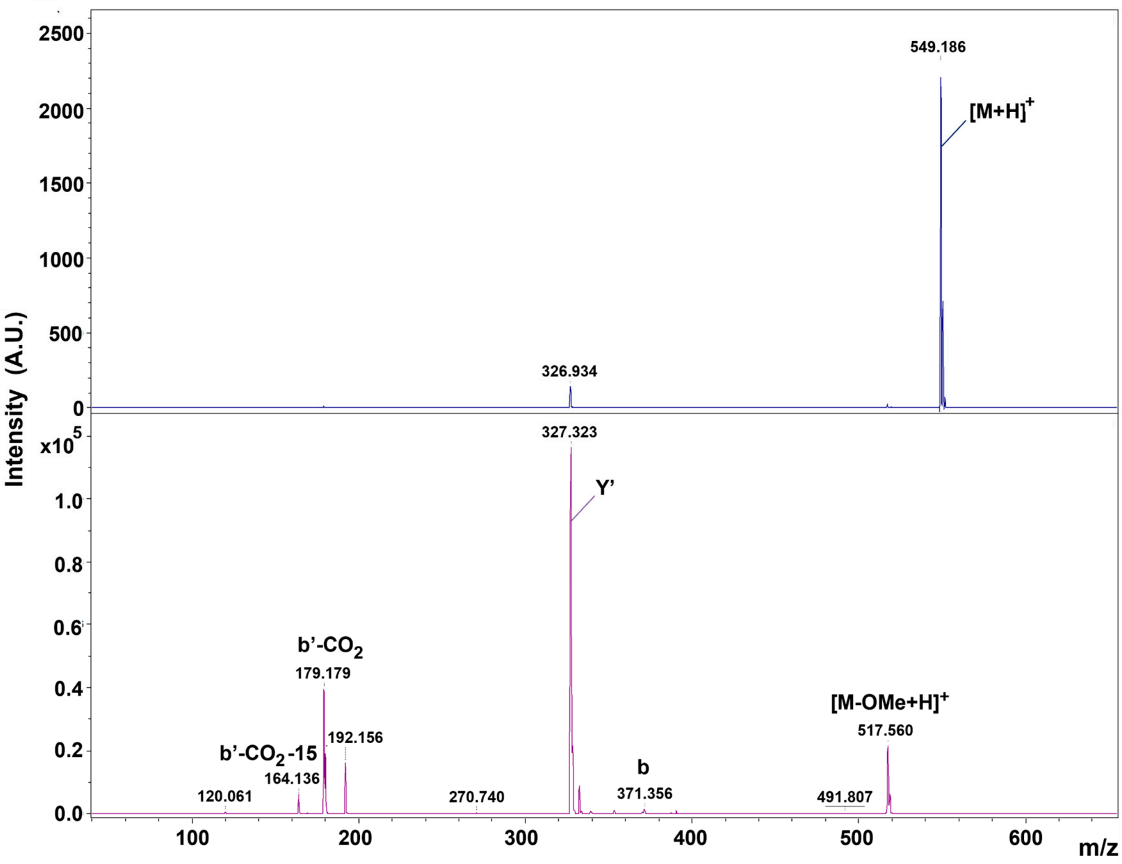

Figure S1. MS/MS spectrum of fragmented protected peptide. The spectrum, originated by partial fragmentation of urethan (Y′) and peptide bond (Y), shows b′-CO_2 ion and most probably its diradical nephew generated by photolysis.

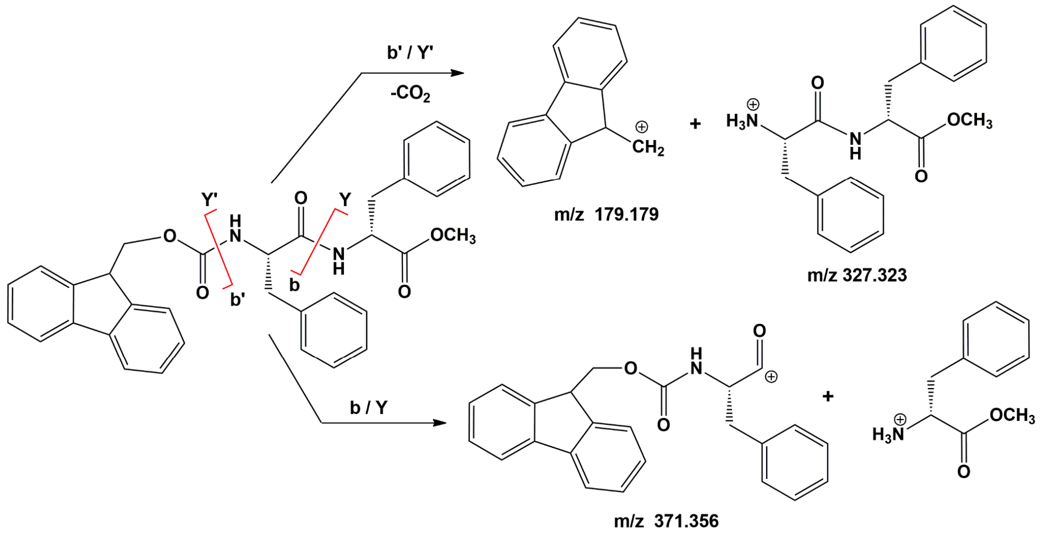

Figure S2. MS/MS Fmoc-Phe-Phe-OMe fragmentation pattern. The ions carrying a charge on the N-terminal fragment are labeled b and b′. The initial b′ ion was unstable and losses a CO_2 molecule generated a much stable fluorenyl carbocation (m/z 179.179). The Y and Y′ ions having a positive charge retained on the C-terminal fragment. Y′ ion has the highest intensity signal in MS/MS spectrum.

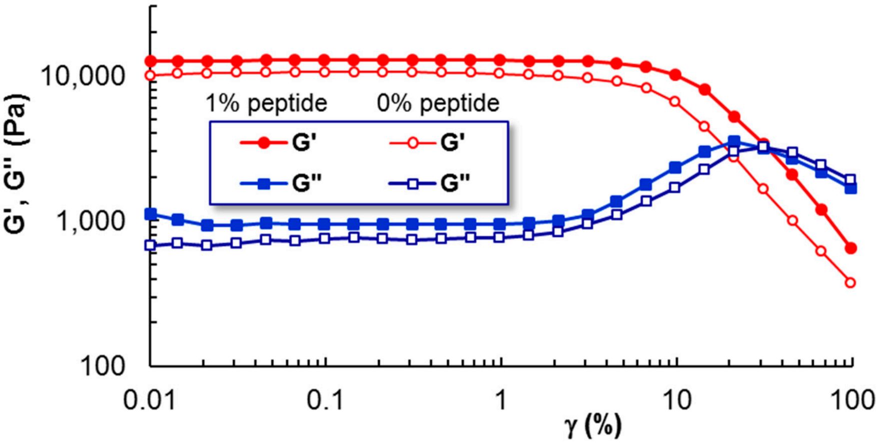

Figure S3. The viscoelastic moduli as a function of strain for 15% PL at 37 °C: full symbols – in the presence of 1% Fmoc-Phe-Phe-OMe; open symbols in the absence of peptide (ω = 1 rad/s).
